# Measurement properties of self-reported outcome measures for older adults with nonspecific low back pain: a systematic review

**DOI:** 10.1093/ageing/afaf045

**Published:** 2025-03-26

**Authors:** Frank F Huang, Jitong Liang, Chung-Ying Lin, Dino Samartzis, Jaro Karppinen, Yongping Zheng, Zhixing Zhou, Daniel K Y Zheng, Jeremy R Chang, Katie de Luca, Arnold Y L Wong

**Affiliations:** Department of Rehabilitation Sciences, The Hong Kong Polytechnic University, Hong Kong SAR, China; Department of Rehabilitation, Henan Provincial People's Hospital, Zhengzhou, Henan, China; Institute of Allied Health Sciences, National Cheng Kung University, Tainan City, Taiwan; Department of Orthopedic Surgery, Rush University Medical Center, Chicago, IL, USA; Medical Research Center Oulu, Oulu University Hospital and University of Oulu, Oulu, Finland; Research Unit of Health Sciences and Technology, University of Oulu, Oulu, Finland; Rehabilitation Services of Wellbeing Services County of South Karelia, Lappeenranta, Finland; Department of Biomedical Engineering, The Hong Kong Polytechnic University, Hong Kong SAR, China; Department of Rehabilitation Sciences, The Hong Kong Polytechnic University, Hong Kong SAR, China; Department of Rehabilitation Sciences, The Hong Kong Polytechnic University, Hong Kong SAR, China; Department of Rehabilitation Sciences, The Hong Kong Polytechnic University, Hong Kong SAR, China; School of Health, Medical and Applied Sciences, CQUniversity, Brisbane, Australia; Department of Rehabilitation Sciences, The Hong Kong Polytechnic University, Hong Kong SAR, China; Research Institute for Smart Ageing, The Hong Kong Polytechnic University, Hong Kong SAR, China

**Keywords:** older adults, nonspecific low back pain, psychometric properties, patient-reported outcome measures, older people, systematic review

## Abstract

**Objective:**

To summarise the measurement properties of patient-reported outcome measures (PROMs) for older adults with nonspecific low back pain.

**Methods:**

Eight databases were searched from inception to January 2024. Two independent reviewers conducted article screening, data extraction, risk of bias assessments, evaluations of measurement properties of PROMs, syntheses of quality of evidence and forming recommendation levels using relevant checklists and assessment tools.

**Results:**

Ten PROMs were identified from 12 included studies. The Functional Rating Index, Oswestry Disability Index, Roland Morris Disability Questionnaire and Quebec Back Pain Disability Scale demonstrated the highest recommendation (category A: PROM most suitable) for evaluating pain-related functional limitation or pain intensity in older adults with acute, subacute or chronic nonspecific low back pain. The Pain Response to Activity and Positioning questionnaire obtained a category A recommendation for making a differential diagnosis of chronic nonspecific low back pain in older adults. The 36-Item World Health Organization Disability Assessment Schedule 2.0 was considered promising (category B: PROM recommended) for assessing physical functioning, while the Back Believe Questionnaire, Catastrophizing Avoidance Scale D-65+, Pain Catastrophizing Scale and Psychological Inflexibility in Pain Scale obtained category B recommendation for evaluating negative thoughts in this population, although further validation is warranted.

**Conclusions:**

This systematic review identified suitable PROMs for assessing physical function in older adults with nonspecific low back pain, but more studies are needed to evaluate the measurement properties of questionnaires on other outcome domains in this population.

## Key Points

- This study summarises the measurement properties of scales for older adults with nonspecific low back pain.
- Ten scales were identified, with four achieving category A for assessing pain-related functional limitations in older adults.
- The Pain Response to Activity and Positioning questionnaire received a category A recommendation for diagnosing this disease.
- The 36-Item WHO Disability Assessment Schedule 2.0 and others received category B.
- There is a need for a multidomain questionnaire to assess the well-being of older individuals with nonspecific low back pain.

## Introduction

Low back pain (LBP) was the most common musculoskeletal disorder, affecting ~619 million individuals globally in 2020 [1]. The prevalence of LBP increases with age, peaking around 85 years [1]. Approximately 90% of individuals with LBP have an unknown aetiology (e.g. epidural abscess, compression fracture, spondyloarthropathy, malignancy, cauda equina syndrome, radicular pain, spinal canal stenosis) [2] and are diagnosed with nonspecific low back pain (NSLBP) [3]. Among older adults, the 12-month prevalence of CLBP (36.1%) [4] was significantly higher than that among individuals aged between 20 and 59 years (ranging from 4.2% to 19.6%) [5]. Given that the global population of individuals aged 65 years or older reached 727 million in 2020 and is predicted to reach 1.5 billion by 2050, effective assessment and management of NSLBP in older adults is crucial to minimise the burdens on these patients and their caregivers globally.

Patient-reported outcome measures (PROMs) are widely used by researchers or clinicians to evaluate and monitor patients’ perceived health status; compare treatment effectiveness; inform clinical decisions; and improve healthcare quality, practices and policies [6]. Recent research [7–9] evaluated the measurement properties (e.g. reliability, validity and responsiveness) of PROMs in assessing the impacts of NSLBP on various core clinical outcomes among working-age adults, such as pain intensity, physical functioning, negative thoughts and health-related quality of life [10]. However, these findings may not generalise to older adults because they may report pain differently due to potential cognitive decline [11] and have different attitudes towards pain and social support [12]. Additionally, older individuals’ social roles, physical conditions, comorbidities and personal goals may differ from those of working-age adults [13–15]. Therefore, it is crucial to determine whether NSLBP-related questionnaires designed for working-age adults demonstrate acceptable measurement properties in the older population.

Although prior studies have reported the validity of using the Oswestry Disability Index (ODI) or Roland-Morris Disability Questionnaire (RMDQ) to evaluate NSLBP-related disability in older adults [16, 17], no systematic review has summarised the psychometric properties of PROMs for evaluating various clinical outcome domains in older adults with NSLBP. Conducting a systematic review of measurement properties (e.g. structural validity, reliability) of scales is an effective method for choosing the most suitable scale to assess a specific disease [18]. A better understanding of the suitability of recommended core outcome measurement instruments for LBP clinical trials [19] among older adults with NSLBP would help clinicians and researchers select appropriate PROMs for this population. This would also facilitate the development or adoption of PROMs to address the strengths and shortcomings of existing tools. Therefore, the current systematic review aimed to summarise the measurement properties of PROMs used for evaluating older people with NSLBP.

## Methods

### Protocol registration and reporting

The current protocol has been registered in PROSPERO. The reporting of the current review followed the Preferred Reporting Items for Systematic Reviews and Meta-Analyses (PRISMA) guidelines [20].

### Literature search

Eight electronic databases, including Cumulative Index to Nursing and Allied Health Literature (CINAHL), Cochrane Library, Medline, Embase, PubMed, PsycINFO, Scopus and SPORTDiscus were searched from their inception to 2 January 2024 to identify relevant articles. Keywords related to ‘measurement properties’, ‘self-reported questionnaires’ ‘older adults’ and ‘low back pain’ were used for searching relevant citations. The complete search strategy is in Supplementary Appendix 1. The measurement property keywords were chosen from the Consensus-Based Standards for the Selection of Health Measurement Instruments (COSMIN) checklist [21] Appropriate Boolean operators were used in the database searches without language restrictions. Search terms were modified for each database to optimise the search. The reference lists of the included articles were examined to identify potentially relevant articles. Forward citation tracking of the included studies was performed using Scopus. The corresponding authors of the included studies were contacted by email to seek additional relevant articles.

### Study criteria

The current review included all study designs if they were full-text peer-reviewed articles evaluating the psychometric properties of self-reported questionnaires for older adults with NSLBP, including acute, subacute and chronic pain. NSLBP was defined as pain or discomfort between the 12th rib and gluteal fold with or without the presence of leg pain [22] and without a known pathoanatomical cause. As the definition of older adults might differ slightly across settings [23–25], studies were eligible for inclusion if they stated that their participants were older adults with NSLBP, and at least 80% of the participants were older adults.

Studies were excluded if there was no statistical data regarding the psychometric properties of self-reported outcome measures for our target population. Furthermore, studies involving participants with specific spinal pathologies (e.g. spinal stenosis, malignancy, trauma, vertebral fracture, infection or inflammatory disorders) were excluded. Additionally, conference abstracts, case reports, systematic reviews, literature reviews, commentaries, editorials, letters to editors or grey literature were excluded.

### Study selection

Citations identified from databases were imported to EndNote 20 (Thomson Reuters, USA) [26] After removing duplicates, two independent reviewers performed pilot title and abstract screening using Rayyan [27]. Any disagreements were discussed and resolved by both reviewers. Persistent disagreements were adjudicated by the third reviewer. After the piloting, the two reviewers independently screened the remaining abstracts using the same procedure. Potential full texts were then retrieved and screened using the same procedure.

### Data extraction

Two independent reviewers extracted relevant data, including authors’ information, year of publication, country, setting, sample size, participants’ demographics, measurement properties [e.g. reliability, internal consistency, content validity, criterion validity, construct validity (i.e. convergent and divergent validity and known group validity) and responsiveness] of identified questionnaires, as well as the relevant statistical results (e.g. root-mean-square error of approximation, Cronbach’s alphas, intraclass correlation coefficients, minimal detectable changes, Pearson’s correlation coefficients and the area under the receiver operating characteristic curve value) [28, 29]. Any between-reviewer disagreements were resolved through discussion with the third reviewer.

### Assessments of methodological quality of included studies

The methodological quality of individual studies was independently appraised by two reviewers using the Quality Appraisal for Clinical Measurement Research Reports Evaluation Form [29]. The details of this assessment are in Supplementary Appendix 1. The overall quality score of each included study was calculated by summing all the item scores, dividing the sum by the number of items and multiplying the results by 100% [29]. Based on the overall score, the quality of the included articles was classified as poor (0%–30%), fair (31%–50%), good (51%–70%), very good (71%–90%) and excellent (>90%) [29].

### Measurement property assessments

Two independent reviewers used the COSMIN Risk of Bias checklist to assess the risk of bias of the included studies that evaluated the measurement properties of PROMs [30]. This checklist has also been used in a similar systematic review [31]. As the identified PROMs might have been adopted from studies involving working-age adults, the PROM development and content validity of each identified PROM were systematically assessed using the initial study that developed the PROM, based on the COSMIN checklist, which includes three separate domains: relevance, comprehensiveness and comprehensibility [32]. Each item in these boxes was graded on a four-point scale as ‘very good’, ‘adequate’, ‘doubtful’ or ‘inadequate’. The overall score for a specific measurement property was determined based on the lowest score among the items within that box [33]. Subsequently, we evaluated each measurement property of a given PROM in an included study against the respective criteria for that property [30]. The results of the included studies were classified as sufficient (+), insufficient (−) or indeterminate (?) for each measurement property [21, 30]. The details of the assessments are in Supplementary Appendix 1.

### Patient-reported outcome measures’ interpretability and feasibility

Interpretability refers to the extent to which qualitative meaning can be attributed, while feasibility is defined as the ease of applying the PROM in its intended context of use, considering constraints such as time or financial limitations [30]. The relevant data were extracted from the included articles.

### Quality of evidence of identified patient-reported outcome measures

The quality of evidence of each psychometric property of a given PROM was assessed using the modified Grading of Recommendations Assessment, Development, and Evaluation (GRADE) approach [21]. The details of this assessment are in Supplementary Appendix 1, and the quality of evidence was rated as high, moderate, low or very low [21].To determine whether a given PROM should be recommended for clinical use, the recommendations were classified into three categories: A, B and C, according to the COSMIN guidelines [21]. Category A means the PROM has the potential to be recommended as the most suitable PROM for the construct and population of interest; the PROM needs to have sufficient content validity (at any level) and at least low-level quality evidence for sufficient internal consistency. Category B indicates that the PROM has the potential to be recommended, but further validation studies are needed (PROM categorised not in A or C). Category C suggests that the PROM should not be recommended because of high-quality evidence for an insufficient measurement property in the PROM.

### Subgroup and sensitivity analyses

The methods of these analyses are in Supplementary Appendix 1.

## Results

### Study selection

A total of 5631 articles were initially identified. After removing duplicates, the remaining 4660 titles and abstracts were screened in Rayyan [27]. Twelve studies [16, 17, 23, 34–42] were included. No additional eligible articles were identified through citation tracking or the corresponding authors. The detailed flow chart of study selection is presented in Supplementary Appendix 1.

### Characteristics of the included studies and identified patient-reported outcome measures

The 12 included studies involved 1536 older participants with NSLBP and were conducted between 2003 and 2023 in eight countries. The number of NSLBP cases in these studies ranged from 26 to 528. Among these studies, three [23, 37, 39] investigated older adults with acute NSLBP (ANSLBP). Seven studies [16, 17, 34–36, 38, 40, 42] involved older individuals with chronic NSLBP (CNSLBP). One study [16] targeted older patients with acute, subacute and chronic NSLBP, while one study [41] examined older individuals with CNSLBP and/or knee pain. The characteristics of these studies are summarised in Table 1, and the details of included PROMs are summarised in Supplementary Appendix 1.

**Table 1:** The characteristics of included studies

Study	Population	Setting	Country	The language version of the PROM	Sample size	Mean age ± SD, age range (year)	Questionnaire	Domain	Interval
Agnieszka *et al*., 2020	Older people with CNSLBP	A hospital	Poland	Polish	65	66.0 ± 11.6, 50 years or older	WHODAS 2.0	Physical functioning	Baseline, 2 days, 1 month
Bayar *et al*., 2003	Older people with CNSLBP	The ageing center registry of community-dwelling older adults in Turkey	Turkey	Turkish	29	76.03 ± 5.89, 65–90	ODI	Physical functioning	Baseline, 7 days
Bayar *et al*., 2004	Older people with CNSLBP	The ageing center registry of community-dwelling older adults from various regions of Turkey	Turkey	English	76	75.76 ± 5.27, 65–90	FRI	Both pain intensity and physical functioning	Baseline, 7 days
de Carvalho *et al*., 2019	Older people with CNSLBP	NS	Brazil	Brazilian	36	71.15 ± 7.23	PRAP	Both pain intensity and physical functioning	Baseline, 3–10 days
Hicks *et al*., 2009	Older people with CNSLBP	The Retirement Community Settings	The United States	English	107	79.6 ± 5.7, 62 years or older	ODI, QBPDS	Physical functioning	Baseline, 11 days (mean number)
Jenk *et al*., 2022	Older people with NSLBP (acute, subacute and chronic)	The clinic of therapists from the Netherlands Chiropractic Association	Netherlands	English	214	66.2 ± 7.8, 55–96	ODI, QBPDS, RMDQ	Physical functioning	Baseline, 2 weeks
Lopes *et al*., 2015	Older people with ANSLBP	A teaching clinic	Brazil	Brazilian	131	55 years or older	PCS	Negative thoughts (pain catastrophising; rumination, magnification n and helplessness)	Baseline, 7–10 days
Nagasawa *et al*., 2021	Older people with CNSLBP and knee pain	A hospital	Japan	Japanese	94	73.8 ± 7.8, 65 years or older	PIPS	Negative thoughts (avoidance of pain and cognitive fusion with pain)	Baseline, 2 weeks
Quint *et al*., 2011	Older people with CNSLBP	NS	Germany	German	68	64 years or older	CAS-D-65+	Negative thoughts (fear avoidance beliefs)	Baseline, 4 weeks
Takara *et al*., 2023	Older people with CNSLBP	NS	Sao Paulo, Brazil	English	528	72 ± 10, 60 years or older	RMDQ	Physical functioning	One single test
Teixeira *et al*., 2020	Older people with ANSLBP	The Department of Physical Therapy of the Universidade Federal de Minas Gerais, Belo Horizonte, Brazil.	Brazil	Brazilian	26	67.4 ± 5.8,60–84	BBQ	Negative thoughts (individual’s beliefs and attitudes about back pain)	Baseline, 1 week
Tingulstad *et al*., 2019	Older people with ANSLBP	At home	Norwegian	Norwegian	116	67.7 ± 8.3, 55 years or older	BBQ	Negative Thoughts (individual’s beliefs and attitudes about back pain)	Baseline, 2 days

Note: ANSLBP, acute nonspecific low back pain; BBQ, Back Beliefs Questionnaire; CAS-D-65+, Catastrophizing Avoidance Scale D-65+; CNSLBP, chronic nonspecific low back pain; FRI, Functional Rating Index; ODI, Oswestry Disability Index; PCS, Pain Catastrophizing Scale; PIPS, Psychological Inflexibility in Pain Scale; PRAP, Pain Response to Activity and Positioning questionnaire; PROM, patient-reported outcome measure; QBPDS, Quebec Back Pain Disability Scale; RMDQ, Roland–Morris Disability Questionnaire; NS, not specified; NSLBP, nonspecific low back pain; WHODAS 2.0, The 36-Item World Health Organization Disability Assessment Schedule 2.0.

### Methodological quality of the included studies and Consensus-Based Standards for the Selection of Health Measurement Instruments risk of bias rating

The methodological quality of the included studies ranged from ‘good’ (54.2%) to ‘excellent’ (95.8%) (Table 3). The most common flaws of the included studies are summarised in Supplementary Appendix 1. The COSMIN risk of bias assessments of the identified 10 PROMs yielded gradings ranging from ‘inadequate’ to ‘very good’ (Table 2). The main reasons for downgrading the reliability of the identified PROMs are summarised in Supplementary Appendix 1.

**Table 2:** Study results and ratings of values on psychometric properties (according to the GRADE approach)

Psychometric property	Instrument	Reference	Risk of Bias	Results (rating)	Quality of evidence (reasons)
**Structural validity**	ODI	Jenk *et al*., 2022	Inadequate	RMSEA = 0.068; SRMR = 0.0588 (+)	Very low
	QBPDS	Jenk *et al*., 2022	Inadequate	CFI = 0.88; TLI = 0.87; RMSEA = 0.18; SRMR = 0.09 (−)	Very low
	RMDQ	Jenk *et al*., 2022	Inadequate	RMSEA = 0.057; SRMR = 0.121 (+)	Low
		Takara *et al*., 2023	Inadequate	RMSEA = 0.037 (+)	
	RMDQ subgroup analysis	Jenk *et al*., 2022	Inadequate	RMSEA = 0.057; SRMR = 0.121 (+)	Very low
	PIPS	Nagasawa *et al*., 2021	Adequate	CFI = 0.97; RMSEA = 0.06 (+)	Low
**Hypothesis testing for construct validity**	ODI	Hicks *et al*., 2009	Doubtful	Evaluating the relationship between ODI and QBPDS in terms of their pain severity and physical function: *P* < .001 (+)	Moderate
		Jenk *et al*., 2022	Very good	ODI met at least 75% of the hypotheses of construct validity (+)	
	ODI subgroup analysis	Jenk *et al*., 2022	Very good	ODI met at least 75% of the hypotheses of construct validity (+)	High
	QBPDS	Hicks *et al*., 2009	Doubtful	Evaluating the relationship between ODI and QBPDS in terms of their pain severity and physical function: *P* < .001 (+)	Moderate
		Jenk *et al*., 2022	Very good	QBPDS met at least 75% of the hypotheses of construct validity (+)	
	QBPDS subgroup analysis	Jenk *et al*., 2022	Very good	QBPDS met at least 75% of the hypotheses of construct validity (+)	High
	RMDQ	Jenk *et al*., 2022	Very good	RMDQ met at least 75% of the hypotheses of construct validity (+)	High
	BBQ	Tingulstad *et al*., 2019	Doubtful	The correlation analysis confirmed 75% of the predefined hypotheses, indicating a good construct validity with FABQ-PA: the correlation coefficient value = −0.57, with PCS: the correlation coefficient value = −0.45, with RMDQ: the correlation coefficient value = −0.45, with NRS: the correlation coefficient value = −0.14 (+)	Low
**Internal consistency**	ODI	Bayar *et al*., 2003	Doubtful	Cronbach’s alpha = 0.722 (baseline)/0.717 (test) (+)	High
		Jenk *et al*., 2022	Very good	Cronbach’s alpha = 0.86 (+)	
	ODI subgroup analysis	Jenk *et al*., 2022	Very good	Cronbach’s alpha = 0.86 (+)	High
	QBPDS	Jenk *et al*., 2022	Very good	Cronbach’s alpha = 0.94 (+)	High
	RMDQ	Bayar *et al*., 2003	Doubtful	Cronbach’s alpha = 0.86 (+)	High
		Jenk *et al*., 2022	Very good	Cronbach’s alpha = 0.89 (+)	
	RMDQ subgroup analysis	Jenk *et al*., 2022	Very good	Cronbach’s alpha = 0.89 (+)	High
	WHODAS 2.0	Agnieszka *et al*., 2020	Doubtful	Cronbach’s alpha = 0.92 (+)	Moderate
	BBQ	Teixeira *et al*., 2020	Inadequate	Cronbach’s alpha = 0.70 (+)	Low
		Tingulstad *et al*., 2019	Doubtful	Cronbach’s alpha = 0.82 (+)	Low
	CAS-D-65+	Quint *et al*., 2011	Doubtful	Cronbach’s alpha = 0.87–0.92 (+)	Moderate
	PIPS	Nagasawa *et al*., 2021	Doubtful	Cronbach’s alpha = 0.85 (+)	Very low
	FRI	Bayar *et al*., 2004	Inadequate	Cronbach’s alpha = 0.921 (test)/0.901 (retest) (+)	Moderate
**Reliability**	ODI	Bayar *et al*., 2003	Doubtful	Test–retest reliability: ICC = 0.93 (+)	Moderate
		Hicks *et al*., 2009	Doubtful	Test–retest reliability: ICC = 0.92 (+)	
		Jenk *et al*., 2022	Adequate	Test–retest reliability: ICC = 0.89 (+)	
	ODI subgroup analysis	Jenk *et al*., 2022	Adequate	Test–retest reliability: ICC = 0.89 (+)	Moderate
	QBPDS	Hicks *et al*., 2009	Doubtful	Test–retest reliability: ICC = 0.92 (+)	Moderate
		Jenk *et al*., 2022	Adequate	Test–retest reliability: ICC = 0.84 (+)	
	QBPDS subgroup analysis	Jenk *et al*., 2022	Adequate	Test–retest reliability: ICC = 0.84 (+)	Moderate
	RMDQ	Jenk *et al*., 2022	Adequate	Test–retest reliability: ICC = 0.85 (+)	Moderate
	WHODAS 2.0	Agnieszka *et al*., 2020	Doubtful	Test–retest reliability: ICC = 0.928 (+)	Very low
	BBQ	Teixeira *et al*., 2020	Inadequate	Test–retest reliability: ICC = 0.74 (+)	Moderate
		Tingulstad *et al*., 2019	Adequate	Test–retest reliability: ICC = 0.71 (+)	
	CAS-D-65+	Quint *et al*., 2011	Inadequate	Test–retest reliability: ICC: 0.67 to 0.70 (−)	Very low
	PCS	Lopes *et al*., 2015	Doubtful	Intra-rater reliability: Kappa = 0.80 ± 0.01, ICC = 0.88 (+)	Low
	FRI	Bayar *et al*., 2004	Doubtful	Test–retest reliability: ICC = 0.913 (+)	Very low
	PRAP	de Carvalho *et al*., 2019	Inadequate	Intra-rater reliability: Kappa = 0.50 to 1.00 (−)	Very low
**Measurement error**	ODI	Jenk *et al*., 2022	Adequate	SDC = 19.11 > MIC = 10 (−)	Moderate
	QBPDS	Jenk *et al*., 2022	Adequate	SDC = 23.58 > MIC = 20 (−)	Moderate
	RMDQ	Jenk *et al*., 2022	Adequate	SDC = 6.87 > MIC = 5 (−)	Moderate
**Criterion validity**	ODI	Bayar *et al*., 2003	Doubtful	Correlation with VAS = 0.53; correlation with RMDQ = 0.66 (−)	Very low
	RMDQ	Bayar *et al*., 2003	Doubtful	Correlation with VAS = 0.46 (−)	Very low
	PIPS	Nagasawa *et al*., 2021	Doubtful	Correlation with acceptance and Action Questionnaire-II = 0.58; Correlation with Cognitive Fusion Questionnaire = 0.45 (−)	Very low
	FRI	Bayar *et al*., 2004	Adequate	Correlation with NRS = 0.701 (+)	Low
**Responsiveness**	ODI	Jenk *et al*., 2022	Very good	AUC = 0.72 (+)	High
	QBPDS	Jenk *et al*., 2022	Very good	AUC = 0.75 (+)	High
	RMDQ	Jenk *et al*., 2022	Very good	AUC = 0.75 (+)	High

Note: AUC, area under the curve; BBQ, Back Beliefs Questionnaire; CAS-D-65+, Catastrophizing Avoidance Scale D-65+; CFA, confirmatory factor analysis; CFI, comparative fit index; CI, confidence interval; CNSLBP, chronic nonspecific low back pain; EFA, exploratory factor analysis; FRI, Functional Rating Index; GFI, goodness of fit index; ICC, Intraclass Correlation Coefficient; KMO, Kaiser–Meyer–Olkin; NNFI = non-normed fit index; NRS, Numeric Rating Scale; NSLBP, nonspecific low back pain; ODI = Oswestry Disability Index; PCS, Pain Catastrophizing Scale; PIPS, Psychological Inflexibility in Pain Scale; PRAP, Pain Response to Activity and Positioning questionnaire; QBPDS, Quebec Back Pain Disability Scale; *r*, Pearson product moment correlation coefficients; RMDQ, Roland-Morris Disability Questionnaire; RMSEA, root-mean-square error of approximation; SDC, smallest detectable change; SRMR, standardised root-mean-square residual; TLI, Tucker–Lewis index; VAS, visual analogue scale; WHODAS 2.0, 36-Item World Health Organization Disability Assessment Schedule 2.0. ‘+’ = Sufficient rating; ‘?’ = Indeterminate rating; ‘−’ = Insufficient rating; ‘±’ = Inconsistent rating; High = High level of confidence in overall ratings; Moderate = Moderate level of confidence in overall ratings; Low = Low level of confidence in overall ratings; Very Low = Very low level of confidence in overall ratings.

**Table 3:** Quality appraisal for clinical measurement research reports evaluation form

Study	Item evaluation criteria^a^												Total (%)	Quality summary
	1	2	3	4	5	6	7	8	9	10	11	12		
Agnieszka *et al*., 2020	2	2	2	2	0	0	2	1	2	1	1	1	66.7	Good
Bayar *et al*., 2003	2	1	2	2	1	2	2	2	2	2	1	2	87.5	Very good
Bayar *et al*., 2004	2	2	2	2	1	0	1	1	2	2	1	2	75	Very good
de Carvalho *et al*., 2019	2	1	2	0	0	1	1	1	2	1	1	1	54.2	Good
Hicks *et al*., 2009	2	2	2	2	1	0	2	2	2	2	2	2	87.5	Very good
Jenk *et al*., 2022	2	2	2	2	1	2	2	2	2	2	2	2	95.8	Excellent
Lopes *et al*., 2015	2	2	2	2	1	0	1	2	2	0	1	2	70.8	Very good
Nagasawa *et al*., 2021	2	2	2	2	2	2	2	2	2	2	1	2	95.8	Excellent
Quint *et al*., 2011	2	2	2	2	1	0	2	2	2	2	0	2	79.2	Very good
Takara *et al*., 2023	2	1	2	2	2	0	2	2	2	2	2	2	87.5	Very good
Teixeira *et al*., 2020	2	2	2	2	1	2	2	1	2	2	2	2	91.7	Excellent
Tingulstad *et al*., 2019	2	2	2	2	1	0	2	2	2	2	2	2	87.5	Very good

Note: Total score = (sum of subtotals/24 × 100%). For a specific paper, if an item is deemed Not Applicable, then, Total score = (sum of subtotals/(2 × number of applicable items) × 100%).

^a^Item evaluation criteria: 1. Thorough literature review to define the research question; 2. Specific inclusion/exclusion criteria; 3.Specific hypotheses; 4. Appropriate scope of psychometric properties; 5. Sample size; 6. Follow-up; 7. The authors referenced specific procedures for administration, scoring and interpretation of procedures; 8. Measurement techniques were standardised; 9. Data were presented for each hypothesis; 10. Appropriate statistics-point estimates; 11. Appropriate statistical error estimates; 12. Valid conclusions and clinical recommendations. The subsections no. 6, asks for percentage of follow-up. This subsection only applies to reliability of test–retest studies. Quality summary: Poor (0%–30%), Fair (31%–50%), Good (51%–70%), Very good (71%–90%) and Excellent (>90%).

**Table 4:** Overall quality of psychometric properties of per instrument

Instrument	Content validity	Structural validity	Construct validity	Internal consistency	Cross-cultural validity	Reliability	Measurement error	Criterion validity	Responsiveness
ODI	+	+	+	+	NR	+	−	−	+
QBPDS	+	−	+	+	NR	+	−	NR	+
RMDQ	+	+	+	+	NR	+	−	−	+
WHODAS 2.0	−	NR	?	+	?	+	NR	NR	?
BBQ	±	NR	+	+	?	+	NR	NR	NR
CAS-D-65+	±	NR	NR	+	?	−	NR	NR	NR
PCS	±	NR	+	NR	?	+	NR	NR	NR
PIPS	±	+	?	+	?	+	NR	−	NR
FRI	+	NR	?	+	NR	+	NR	+	NR
PRAP	+	NR	NR	NR	?	−	NR	NR	NR

Note: The overall quality of psychometric properties (apart from the content validity) was rated using the criteria for good psychometric properties [30]; ‘+’ = sufficient rating; ‘−’ = insufficient rating; ‘?’ = indeterminate rating (due to less robust psychometric data); ‘±’ = inconsistent rating. Abbreviations: BBQ, Back Beliefs Questionnaire; CAS-D-65+, Catastrophizing Avoidance Scale D-65+; FRI, Functional Rating Index; NR, not reported; ODI, Oswestry Disability Index; PCS, Pain Catastrophizing Scale; PIPS, Psychological Inflexibility in Pain Scale; PRAP, Pain Response to Activity and Positioning questionnaire; QBPDS, Quebec Back Pain Disability Scale; RMDQ, Roland-Morris Disability Questionnaire; WHODAS 2.0, The 36-Item World Health Organization Disability Assessment Schedule 2.0.

### Measurement properties

#### Content validity

None of the included studies reported the content validity of the identified PROMs.

The content validity of all the PROM development studies, except for Catastrophizing Avoidance Scale D-65+ (CAS-D-65+) [36], was rated as indeterminate [43–51]. The rating of CAS-D-65+ was significantly affected by the absence of the original study that detailed the development of this PROM. Consequently, we considered the PROM development studies, the included studies and the ratings provided by reviewers to determine the overall ratings for each PROM. In terms of physical functioning, there is low-quality evidence supporting adequate content validity of ODI [16, 17, 34, 43], Quebec Back Pain Disability Scale (QBPDS) [16, 17, 46] and RMDQ [16, 42, 44] in measuring CNSLBP-related disability. However, the 36-Item World Health Organization Disability Assessment Schedule 2.0 (WHODAS 2.0) [40, 51] was considered to have inadequate content validity with low-quality evidence.

In terms of negative thoughts, low-quality evidence suggested that the Back Beliefs Questionnaire (BBQ) [37, 39, 47], CAS-D-65+ [36] and Psychological Inflexibility in Pain Scale (PIPS) [41, 50] had inconsistent content validity in evaluating various negative thoughts. However, very low-quality evidence showed that Pain Catastrophizing Scale (PCS) [23, 45] also had inconsistent content validity in assessing pain catastrophising. Regarding the assessments of both pain intensity and physical functioning, low-quality evidence supported the adequate content validity of FRI [35, 49]. There was low-quality evidence supporting that PRAP had adequate content validity for diagnosing CNSLBP in older adults [38, 48]. The details regarding the content validity of the included PROMs are presented in Supplementary Appendix 2.

#### Internal consistency

High-quality evidence supported the sufficient internal consistency of ODI [16, 34], QBPDS [16] and RMDQ [16, 34] for assessing NSLBP-related disability in older adults with acute, subacute and chronic NSLBP. Additionally, there was moderate-quality evidence supporting the sufficient internal consistency of WHODAS 2.0 for assessing NSLBP-related disability in older adults with CNSLBP [40].

There was low-quality evidence indicating that BBQ had sufficient internal consistency for evaluating negative thoughts in older adults with ANSLBP [39]. Likewise, there was moderate- and very low-quality evidence supporting the sufficient internal consistency of CAS-D-65+ [36] and PIPS [41], respectively. Additionally, FRI demonstrated sufficient internal consistency for measuring both pain intensity and physical functioning in older adults with CNSLBP, with moderate-quality evidence [35]. The Cronbach’s alpha value for internal consistency in these scales ranged from 0.70 to 0.94. However, no included studies evaluated the internal consistency of PCS or PRAP in older adults (Tables 2 and 4).

#### Subgroup and sensitivity analyses

The results of these analyses are shown in Supplementary Appendix 1.

### Recommendations for usage

The recommendations regarding the suitability of the 10 identified PROMs for use in older adults with NSLBP are shown in Table 5. Notably, ODI [16, 17], QBPDS [16, 17] and RMDQ [16, 42] were rated as appropriate tools (category A) for evaluating physical functioning in older adults with acute, subacute or chronic NSLBP. Additionally, FRI [35] was categorised as ‘A’ for evaluating both pain intensity and physical functioning in older individuals with CNSLBP. PRAP [38] was also classified as a suitable tool (category A) for diagnosing CNSLBP in older adults.

**Table 5:** Recommendations on suitable instruments for their future use

Category	Description on category (criteria)	Instruments
A: Most suitable	Instruments with evidence for sufficient content validity (any level) AND at least low-level quality evidence for sufficient internal consistency. Instruments can be recommended for use, and results obtained with these instruments can be trusted.	ODI, QBPDS, RMDQ, FRI, PRAP
B: Promising but need further validation	Instruments have potential to be recommended for use, but they require further research to assess the quality of these (instrument categorised not in A or C).	WHODAS 2.0, BBQ, CAS-D-65+, PCS, PIPS
C: Not recommendable	Instruments with high-quality evidence for an insufficient measurement property and should not be recommended for use.	

Note: BBQ, Back Beliefs Questionnaire; CAS-D-65 +, Catastrophizing Avoidance Scale D-65+; FRI, Functional Rating Index; ODI, Oswestry Disability Index; PCS, Pain Catastrophizing Scale; PIPS, Psychological Inflexibility in Pain Scale; PRAP, Pain Response to Activity and Positioning questionnaire; QBPDS, Quebec Back Pain Disability Scale; RMDQ, Roland–Morris Disability Questionnaire; WHODAS 2.0, The 36-Item World Health Organization Disability Assessment Schedule 2.0.

WHODAS 2.0 [40] had the potential for evaluating physical functioning in older adults with CNSLBP (category B), while BBQ [37, 39], CAS-D-65+ [36], PCS [23] and PIPS [41] showed promise for evaluating negative thoughts in older adults with acute or chronic NSLBP (category B), although further validation is warranted.

## Discussion

This is the first systematic review to summarise the measurement properties of PROMs for assessing older adults with NSLBP. While ODI, QBPDS and RMDQ are recommended for assessing physical functioning in this population, FRI is a recommended composite questionnaire for evaluating pain intensity and physical functioning. PRAP is recommended for diagnosing CNSLBP in older adults. WHODAS 2.0 and four other negative thought questionnaires (BBQ, CAS-D-65+, PCS and PIPS) require further validation in older adults with NSLBP before being recommended for clinical practice/research.

### Clinical implications

Our review offers valuable insights into the use of PROMs for assessing pain intensity, physical functioning and negative thoughts related to NSLBP in older adults. Previous research has recommended the use of Numeric Rating Scale (NRS) as a standard for assessing pain intensity in individuals with NSLBP [10]. However, none of the included studies evaluated the measurement properties of using NRS in older adults with NSLBP. Considering the widespread use of NRS for evaluating pain intensity in older adults with various pain conditions (e.g. postoperative pain, knee pain or back pain) in prior research [52–54], it is reasonable to assume that NRS is a valid tool for assessing pain intensity in older adults with NSLBP.

The FRI [35] obtained a category A recommendation for assessing both pain intensity and physical functioning in older adults with CNSLBP. However, further research is needed to compare this scale with NRS in assessing pain intensity in older adults because its construct validity in relation to NRS is unknown.

The PRAP [38] obtained a category A recommendation in the diagnosis of CNSLBP by assessing the performance of various daily activities using a four-level pain intensity scale. [48]. However, as the study that investigated the PRAP [38] only assessed the Brazilian version of the scale, additional validation of this scale in other languages is warranted to diagnose NSLBP in older adults across different countries.

The ODI [16, 34], QBPDS [16, 17] and RMDQ [16, 42] received a category A recommendation for evaluating physical functioning in older adults with NSLBP because of their sufficient content validity and internal consistency [16]. Among these three scales, the QBPDS [16] exhibited insufficient structural validity. However, according to the COSMIN guideline for PROM recommendations [21] (Table 5), the QBPDS was classified as category A due to its sufficient content validity and internal consistency. Given that sufficient structural validity is essential to ensure that the scores obtained from a PROM accurately reflect the underlying dimensions or structure of the construct being measured [55], further research is needed to establish the structural validity of QBPDS.

WHODAS 2.0 has received a category B recommendation for measuring general functioning and disability across various domains, including cognition, mobility, self-care, getting along, life activities and participation [51]. However, it may lack the relevance (content validity) to specifically assess NSLBP-related disability in older adults with chronic NSLBP. Therefore, we suggest the ODI and RMDQ as the preferred PROMs for evaluating NSLBP-related disability in older adults with NSLBP.

None of the identified PROMs that assess negative thoughts (e.g. false beliefs or pain catastrophising) in older adults with NSLBP received a category A recommendation due to insufficient content validity. Therefore, there is a need for high-quality scales specifically designed to evaluate negative thoughts in older adults with NSLBP, as NSLBP may lead to negative psychological problems (e.g. depression). Future studies should evaluate the measurement properties of other relevant PROMs that evaluate various psychological issues in older adults with NSLBP.

Collectively, the current evidence supports the use of several existing PROMs for evaluating the physical and psychological well-being of older adults with NSLBP. However, it may be time-consuming and challenging for older adults to complete multiple PROMs to comprehensively evaluate the impacts of NSLBP. Future research should explore the necessity of developing or adopting a multidomain questionnaire to effectively assess the biopsychosocial well-being of older individuals with NSLBP.

### Strengths and limitations

The current review has several strengths. First, the study protocol was registered with PROSPERO enhancing transparency and followed the PRISMA guidelines for reporting. Second, comprehensive database searches, standardised screening and data extraction procedures and established tools were employed to evaluate the measurement properties of identified PROMs and the included studies. Third, a modified GRADE approach was used to synthesise the evidence.

This review has some limitations. Firstly, no universal definition of ‘older adults’ exists. While most studies included in this review defined older adults as individuals aged 60 or 65 years and above [56], one study defined older adults as individuals aged 55 years or older [23, 24]. This discrepancy may have contributed to the variability of our findings. Additionally, most of the identified tools (e.g. WHODAS 2.0, CAS-D-65+, PCS and PRAP) were only validated in older adults in a single country (e.g. Korea, Japan, Poland) and specific settings (e.g. hospitals). Therefore, the recommendation of the PROMs needs future robust evidence to corroborate, especially cross-country evidence.

## Conclusions

This systematic review is the first to provide a comprehensive summary of the evidence on the measurement properties of PROMs for assessing the impacts of NSLBP on older adults. The ODI, QBPDS and RMDQ had category A recommendations for evaluating NSLBP-related disability in older adults with NSLBP. FRI had a category A recommendation for evaluating both pain intensity and physical functioning in older adults with CNSLBP. Additionally, PRAP was rated as a category A recommendation for making a differential diagnosis of CNSLBP in older adults. The WHODAS 2.0 had a category B recommendation and requires further validation, while the BBQ, CAS-D-65+, PCS and PIPS were potentially useful for assessing negative thoughts in older adults with NSLBP, with a category B recommendation. However, the content validity of these PROMs for assessing older adults with NSLBP remains indeterminate based on the findings of their respective PROM development studies. Future research should address their content validity and explore the necessity of developing a multidomain questionnaire for comprehensive evaluation of the impacts of NSLBP on older adults.

## Supplementary Material

aa-24-1148-File003_afaf045

aa-24-1148-File004_afaf045
