# Supplementary material for: Measurement properties of self-reported outcome measures for older adults with nonspecific low back pain: a systematic review
Source: Age Ageing. 2025 Mar 26;54(3):afaf045. doi: 10.1093/ageing/afaf045 (PMC11942786; doi:10.1093/ageing/afaf045)

**Appendix 1.**

**Measurement properties of self-reported outcome measures for older adults with non-specific low back pain: a systematic review**

[Supplementary text 2](#_Toc186995263)

1 [Supplementary methods 3](#_Toc186995264)

[1.1 Evaluation criteria of the Quality Appraisal for Clinical Measurement Research Reports Evaluation Form 3](#_Toc186995265)

[1.2 Consensus-Based Standards for the Selection of Health Measurement Instruments Risk of Bias checklist 3](#_Toc186995266)

[1.3 The modified Grading of Recommendations Assessment, Development, and Evaluation (GRADE) approach 4](#_Toc186995267)

[1.4 Subgroup and sensitivity analyses 4](#_Toc186995268)

2 [Supplementary results 5](#_Toc186995269)

[2.1 Characteristics of the included studies and identified PROMs 5](#_Toc186995270)

[2.2 Methodological quality of the included studies and COSMIN risk of bias rating 6](#_Toc186995271)

[2.3 Measurement properties 7](#_Toc186995272)

[2.3.1 Structural validity 7](#_Toc186995273)

[2.3.2 Hypotheses testing for construct validity 8](#_Toc186995274)

[2.3.3 Cross-cultural validity 9](#_Toc186995275)

[2.3.4 Reliability 9](#_Toc186995276)

[2.3.5 Measurement errors 11](#_Toc186995277)

[2.3.6 Criterion validity 11](#_Toc186995278)

[2.3.7 Responsiveness 11](#_Toc186995279)

[2.3.8 Interpretability and feasibility of identified PROMs 12](#_Toc186995280)

[2.3.9 Subgroup and sensitive analyses 12](#_Toc186995281)

3 [Supplementary discussion 13](#_Toc186995282)

[3.1 Psychometric properties of identified questionnaires 13](#_Toc186995283)

[Reference 16](#_Toc186995284)

[Supplementary tables 20](#_Toc186995285)

[Supplementary Table 1. Search Strategy. 20](#_Toc186995286)

[Supplementary Table 2. Consensus-based Standards for the selection of health Measurement Instruments criteria and rating system for evaluating the content validity of instruments adapted from Mokkink et al. (2018). 22](#_Toc186995287)

[Supplementary Table 3. Criteria for Good Psychometric Properties Adapted from Prinsen et al. (2018). 24](#_Toc186995288)

[Supplementary Table 4. Study results and ratings of values on psychometric properties (according to GRADE approach). 27](#_Toc186995289)

[Supplementary Table 5. Details of Consensus-based Standards for the selection of health Measurement Instruments Risk of Bias. 37](#_Toc186995290)

[Supplementary Table 6. Overall quality of content validity and quality of evidence per instrument. 41](#_Toc186995291)

[Supplementary Fig.1. A PRISMA flow diagram of the study selection process. 43](#_Toc186995292)

**Supplementary text**

**1 Supplementary methods**

**1.1 Evaluation criteria of the Quality Appraisal for Clinical Measurement Research Reports Evaluation Form**

This appraisal tool consists of 12 evaluation criteria, including thorough literature review to define the research question, specific inclusion or exclusion criteria, specific hypotheses, appropriate scope of psychometric properties, sample size, follow-up, the authors’ referenced specific actions for administration, scoring and interpretation of procedures, standardized measurement techniques; presentation of data for each hypothesis, appropriate statistics-point estimates, appropriate statistical error estimates, and valid conclusions and recommendations [1].

**1.2 Consensus-Based Standards for the Selection of Health Measurement Instruments Risk of Bias checklist**

Consensus-Based Standards for the Selection of Health Measurement Instruments Risk of Bias checklist focuses on the reliability and validity of the instruments, consisting of 10 measurement property boxes (namely PROM development, content validity, structural validity, hypotheses testing for construct validity, internal consistency, cross-cultural validity, reliability, measurement error, criterion validity, responsiveness).

**1.3 The modified Grading of Recommendations Assessment, Development, and Evaluation (GRADE) approach**

This approach takes into account four domains: the risk of bias of the included studies [as determined by the Consensus-Based Standards for the Selection of Health Measurement Instruments (COSMIN) Risk of Bias score for each measurement property]; inconsistency (unexplained inconsistency of results across studies); imprecision (total sample size in each scale of the included studies); and indirectness (evidence from populations different from the population of interest in the review) [2].

## **1.4 Subgroup and sensitivity analyses**

Subgroup analyses were conducted based on the age of older individuals and the duration of NSLBP. Specifically, we performed a subgroup analysis on the youngest-old (65-74 years), middle-old (75-84 years), and oldest-old (over 85 years) age groups [3]. Likewise, a subgroup analysis was conducted based on the acute (< four weeks), subacute (four to 12 weeks), and chronic (>12 weeks) stages of NSLBP [4]. Furthermore, a sensitivity analysis was performed to evaluate the effect of excluding low-quality papers on our conclusions [5]. If there was inadequate data to conduct subgroup and sensitivity analyses, the analysis would not be carried out.

**2 Supplementary results**

**2.1 Characteristics of the included studies and identified PROMs**

Ten PROMs were identified from these studies. Four of them evaluated physical functioning, including ODI [6–8], Quebec Back Pain Disability Scale (QBPDS) [7,8], RMDQ [8], and the 36-Item World Health Organization Disability Assessment Schedule 2.0 (WHODAS 2.0) [9]. Another four PROMs were developed specifically to assess negative thoughts of older adults with NSLBP, namely the back believe questionnaire (BBQ) [10,11], Catastrophizing Avoidance Scale D-65+ (CAS-D-65+) [12], Pain Catastrophizing Scale (PCS) [13], and Psychological Inflexibility in Pain Scale (PIPS) [14]. Functional Rating Index (FRI) [15] was employed to assess both pain intensity and physical functioning for older adults with CNSLBP, while Pain Response to Activity and Positioning questionnaire (PRAP) [16] was utilized for diagnosing CNSLBP in older adults by evaluating the self-reported performance of daily activities. The reported psychometric properties of these questionnaires are summarized in Table 2 by using the GRADE [2] approach. (More detailed scoring steps of Table 2 were shown in Supplementary Table 4).

Ten PROMs were identified from these studies. Four of them evaluated physical functioning, including ODI [6–8], Quebec Back Pain Disability Scale (QBPDS) [7,8], RMDQ [8], and the 36-Item World Health Organization Disability Assessment Schedule 2.0 (WHODAS 2.0) [9]. Another four PROMs were developed specifically to assess negative thoughts of older adults with NSLBP, namely the back believe questionnaire (BBQ) [10,11], Catastrophizing Avoidance Scale D-65+ (CAS-D-65+) [12], Pain Catastrophizing Scale (PCS) [13], and Psychological Inflexibility in Pain Scale (PIPS) [14]. Functional Rating Index (FRI) [15] was employed to assess both pain intensity and physical functioning for older adults with CNSLBP, while Pain Response to Activity and Positioning questionnaire (PRAP) [16] was utilized for diagnosing CNSLBP in older adults by evaluating the self-reported performance of daily activities. The reported psychometric properties of these questionnaires are summarized in Table 2 by using the GRADE [2] approach. (More detailed scoring steps of Table 2 were shown in Supplementary Table 4.)

## **2.2 Methodological quality of the included studies and COSMIN risk of bias rating**

The methodological quality of the included studies ranged from “good” (54.2%) to “excellent” (95.8%) (Table 3). The most common flaws of the included studies for evaluating the reliability of PROMs were the absence of sample size calculations, missing data at follow-up, and failure to provide appropriate statistical error estimates. The COSMIN risk of bias assessments of the identified 10 PROMs yielded gradings ranging from “inadequate” to “very good” (Table 2 and Supplementary Table 5). The main reasons for downgrading the reliability of the identified PROMs were unclear allocation of participants, unknown stability of the construct being measured during the interim period, and inadequate time interval between tests.

## **2.3 Measurement properties**

### 2.3.1 Structural validity

For physical functioning, there was very low-quality evidence supporting the sufficient structural validity of the ODI [8] for assessing older adults with acute, subacute, and chronic NSLBP (RMSEA=0.068; SRMR=0.0588) (Tables 2 and 4 in manuscript). Additionally, low-quality and very low-quality evidence supported the sufficient structural validity of the RMDQ for older adults with CNSLBP and acute or subacute NSLBP, respectively. [8,17]. However, very low-quality evidence indicated that the QBPDS (CFI=0.88; TLI=0.87; RMSEA=0.18; SRMR=0.09) had insufficient structural validity for evaluating older individuals with acute, subacute, and chronic NSLBP [7,8]. No included studies examined the structural validity of WHODAS 2.0 in older adults.

Regarding negative thoughts, low-quality evidence supported the sufficient structural validity of PIPS for evaluating older adults with CNSLBP (CFI=0.97; RMSEA=0.06) [18]. However, the structural validity of BBQ, CAS-D-65+, PCS, FRI, and PRAP in older adults with NSLBP was not examined (Tables 2 and 4 in manuscript).

### 2.3.2 Hypotheses testing for construct validity

The construct validity of three physical functioning questionnaires, namely ODI, QBPDS and RMDQ, was evaluated through testing a priori hypotheses, and the results confirmed the hypothesized associations with demographic factors, physical tests, pain, and health-related quality of life) [8]. Furthermore, one included study [7] investigated the construct validity of the ODI and QBPDS by evaluating the association between pain severity and physical function scores, although no a priori hypothesis was provided. Overall, there is moderate-quality evidence supporting the construct validity of the ODI and QBPDS, while high-quality evidence substantiates the construct validity of the RMDQ. The construct validity of the WHODAS 2.0 [9] was considered indeterminate because no a priori hypotheses were mentioned in the evaluation of its construct validity.

For negative thoughts, there was low-quality evidence indicating that the BBQ had sufficient construct validity for assessing older adults with ANSLBP because three out of the four proposed hypotheses were confirmed by examining the correlations between the absolute values of the BBQ and the Fear-Avoidance Beliefs Questionnaire for physical activity, PCS, or RMDQ [10]. The construct validity of the PIPS [14] and FRI [15] was considered indeterminate because no a priori hypotheses were mentioned in testing their construct validity. The construct validity of the CAS-D-65+, PCS, and PRAP were not investigated (Tables 2 and 4 in manuscript).

### 2.3.3 Cross-cultural validity

The cross-cultural validity of the WHODAS 2.0 [9], BBQ [10,11], CAS-D-65+ [12], PCS [13], PIPS [14], and PRAP [16] was considered indeterminate because the relevant studies did not perform multiple group factor analysis or differential item functioning analysis to assess cross-cultural validity. No included studies investigated the cross-cultural validity of the remaining PROMs (Tables 2 and 4 in manuscript).

### 2.3.4 Reliability

For physical functioning, there was moderate-quality evidence supporting the sufficient test-retest reliability of the ODI [6–8], QBPDS [7,8] and RMDQ [8] in older adults with acute, subacute, and chronic NSLBP. Similarly, very low-quality evidence supported the sufficient test-retest reliability of WHODAS 2.0 in assessing old individuals with CNSLBP [9].

For negative thoughts, the BBQ [10,11] and PCS [13] demonstrated sufficient test-retest reliability in older individuals with ANSLBP, supported by moderate- and low-quality evidence, respectively. However, there was very low-quality evidence that CAS-D-65+ had insufficient test-retest reliability when assessing older adults with CNSLBP [12].

Moderate-quality evidence suggested that the FRI had sufficient test-retest reliability for assessing pain intensity and physical functioning in older adults with CNSLBP [15], whereas low-quality evidence indicated that PRAP [16] had insufficient test-retest reliability in diagnosing CNSLBP in older adults based on their performance of daily activities. No included study investigated the test-retest reliability of PIPS in older adults with NSLBP (Tables 2 and 4 in manuscript).

###

### 2.3.5 Measurement errors

Moderate-quality evidence indicated that the ODI, QBPDS, and RMDQ [8] had insufficient measurement errors. No included studies evaluated the measurement errors of other identified PROMs (Tables 2 and 4 in manuscript).

### 2.3.6 Criterion validity

There was very low-quality evidence that the criterion validity of the ODI [6], RMDQ [6] and PIPS [14] was insufficient when measured by concurrent validity in older adults with CNSLBP. However, low-quality evidence supported the sufficient concurrent validity of FRI [15] in older adults with CNSLBP, as verified by comparing responses to the FRI with the results of the Numeric Rating Scale (NRS). The criterion validity of other identified PROMs was not investigated in older adults (Tables 2 and 4 in manuscript).

### 2.3.7 Responsiveness

High-quality evidence corroborated the sufficient responsiveness of the ODI, QBPDS, and RMDQ [8]. Conversely, there was very low-quality evidence that WHODAS 2.0 [9] had an indeterminate responsiveness. The responsiveness of other identified PROMs was not investigated in any of the included studies (Tables 2 and 4 in manuscript).

### 2.3.8 Interpretability and feasibility of identified PROMs

Given the lack of relevant data from the included studies, the interpretability and feasibility of the identified PROMs remain unknown.

### 2.3.9 Subgroup and sensitive analyses

Due to the lack of relevant data regarding the youngest-old, middle-old, and oldest-old age groups in the included studies, the planned age subgroup analysis was not conducted. However, the subgroup analyses of various measurement properties (e.g., content validity, structural validity, construct validity, internal consistency, reliability) of the ODI, QBPDS, and RMDQ were performed based on the chronicity of NSLBP because one included study reported the properties of these PROMs in older adults with acute, subacute and chronic NSLBP [8]. However, since three other studies included in this review primarily evaluated the measurement properties of these three scales in older adults with CNSLBP [6,7,17], these studies were excluded from the analysis of measurement properties in older adults with acute or subacute NSLBP. Overall, these three scales showed adequate content validity for assessing older adults with acute, subacute, or chronic NSLBP (Appendix A and Supplementary Table 5). Therefore, the ODI, QBPDS, and RMDQ yielded a category A recommendation for assessing NSLBP-related disability in older adults with acute, subacute, or chronic NSLBP. The subgroup analysis results of other measurement properties of these three scales are shown in Table 2 of the manuscript.

No sensitive analysis was conducted in this review because all the included studies demonstrated good to excellent methodological quality (Table 3 in manuscript).

## **3 Supplementary** **discussion**

### 3.1 Psychometric properties of identified questionnaires

Content validity significantly affects the psychometric properties and recommendation level of PROMs [19] Our ad-hoc analyses found varying levels of content validity (i.e., relevance, comprehensiveness, and comprehensibility) for the included PROMs, ranging from “inconsistent” to “sufficient”. The ODI, QBPDS, RMDQ, FRI and PRAP demonstrated sufficient content validity. However, other scales exhibited inconsistent or insufficient content validity due to certain limitations. Specifically, WHODAS 2.0 [20] lacked relevance to the NSLBP population in assessing physical functioning in older adults with CNSLBP. Likewise, BBQ did not include older adults with NSLBP in its development study [21]. The study that developed CAS-D-65+ [12] could not be found. In the case of PCS, which evaluated pain catastrophizing in older adults with ANSLBP [13], there was no patient or expert group interviews, nor the involvement of older adults with NSLBP in its development [22]. Furthermore, PCS [22,13] and PlPS [23,14] exhibited discrepancies in target groups between the development and the included articles. These findings highlight the need for further validation in future studies.

The structural validity of certain identified PROMs is uncertain. According to the COSMIN guideline, PROMs with insufficient structural validity require further evaluation [24]. The ODI, RMDQ, and PIPS possess sufficient structural validity, while the QBPDS is deemed insufficient in this domain, It is worth noting that the studies included in this review [6,12,10,9,11] did not evaluate the structural validity of WHODAS 2.0, BBQ, CAS-D-65+, PCS, FRI, or PRAP, despite their satisfactory internal consistency indicated by Cronbach’s alpha values. Future studies should validate the structural validity of these questionnaires in older adults with NSLBP.

The cross-cultural validity of the identified questionnaires is uncertain. Although some of the included studies used translated versions of WHODAS 2.0, BBQ, CAS-D-65, PCS, PIPS, and PRAP to evaluate older adults with NSLBP, none of them evaluated their cross-cultural validity in this population. This limitation hampers the applicability of these questionnaires in global clinical research or practice [25].

most of the identified PROMs did not evaluate their criterion validity in older adults with NSLBP. Only the FRI [15] demonstrated sufficient criterion validity. The absence of comparisons may be attributed to the absence of a universally accepted “gold standard” measurement for each outcome domain (e.g., pain intensity, physical functioning, and negative thoughts) in older adults with NSLBP. To address this issue, it is recommended to establish an international consortium dedicated to determining the “gold standard” for measuring each outcome domain through Delphi research and relevant psychometric property studies.

The measurement properties of several identified PROMs were comparable to those reported in other age groups. Specifically, the reported reliability and validity of ODI [6–8], QBPDS [6–8], RMDQ [8,17], and PCS [13] in our included studies were similar to the corresponding measurement properties of these questionnaires in general population with NSLBP [26–33]. For instance, the moderate to high correlations of reliability and validity observed between the ODI or RMDQ and the Visual Analogue Scale in the older population [6] were similar to those observed in studies involving participants of all age groups [34–38].

### Reference

1. Law MC, MacDermid J. *Evidence-Based Rehabilitation: A Guide to Practice*. Thorofare, NJ: Slack Incorporated, 2014.

2. Prinsen CAC, Mokkink LB, Bouter LM *et al.* COSMIN guideline for systematic reviews of patient-reported outcome measures. *Qual Life Res Int J Qual Life Asp Treat Care Rehabil* 2018;**27**:1147–57.

3. Alterovitz SSR, Mendelsohn GA. Relationship goals of middle-aged, young-old, and old-old Internet daters: an analysis of online personal ads. *J Aging Stud* 2013;**27**:159–65.

4. Qaseem A, Wilt TJ, McLean RM *et al.* Noninvasive Treatments for Acute, Subacute, and Chronic Low Back Pain: A Clinical Practice Guideline From the American College of Physicians. *Ann Intern Med* 2017;**166**:514.

5. Mokkink LB, de Vet HCW, Prinsen C a. C *et al.* COSMIN Risk of Bias checklist for systematic reviews of Patient-Reported Outcome Measures. *Qual Life Res Int J Qual Life Asp Treat Care Rehabil* 2018;**27**:1171–9.

6. Bayar K, Bayar B, Yakut E *et al.* Reliability and construct validity of the Oswestry Low Back Pain Disability Questionnaire in the elderly with low back pain. *Pain Clin* 2003;**15**:55–9.

7. Hicks GE, Manal TJ. Psychometric properties of commonly used low back disability questionnaires: are they useful for older adults with low back pain? *Pain Med Malden Mass* 2009;**10**:85–94.

8. Jenks A, Hoekstra T, van Tulder M *et al.* Roland-Morris Disability Questionnaire, Oswestry Disability Index, and Quebec Back Pain Disability Scale: Which Has Superior Measurement Properties in Older Adults With Low Back Pain? *J Orthop Sports Phys Ther* 2022;**52**:457–69.

9. Ćwirlej-Sozańska A, Bejer A, Wiśniowska-Szurlej A *et al.* Psychometric Properties of the Polish Version of the 36-Item WHODAS 2.0 in Patients with Low Back Pain. *Int J Environ Res Public Health* 2020;**17**:7284.

10. Tingulstad A, Munk R, Grotle M *et al.* Back beliefs among elderly seeking health care due to back pain; psychometric properties of the Norwegian version of the back beliefs questionnaire. *BMC Musculoskelet Disord* 2019;**20**:510.

11. Teixeira LF, Diz JBM, Silva SLA da *et al.* Cross-cultural adaptation, validity and reproducibility of the Back Beliefs Questionnaire among older Brazilians with acute low back pain. A cross-sectional study. *Sao Paulo Med J Rev Paul Med* 2020;**138**:287–96.

12. Quint S, Raich M, Luckmann J. [Evaluation of a two-dimensional scale for the assessment of fear avoidance beliefs in elderly chronic low back pain patients]. *Schmerz Berl Ger* 2011;**25**:315–21.

13. Lopes RA, Dias RC, Queiroz BZ de *et al.* Psychometric properties of the Brazilian version of the Pain Catastrophizing Scale for acute low back pain. *Arq Neuropsiquiatr* 2015;**73**:436–44.

14. Nagasawa Y, Shibata A, Fukamachi H *et al.* The Psychological Inflexibility in Pain Scale (PIPS): Validity and Reliability of the Japanese Version for Chronic Low Back Pain and Knee Pain. *J Pain Res* 2021;**14**:325–32.

15. Bayar B, Bayar K, Yakut E *et al.* Reliability and validity of the Functional Rating Index in older people with low back pain: preliminary report. *Aging Clin Exp Res* 2004;**16**:49–52.

16. de Carvalho GR, de Oliveira EA, Rocha VTM *et al.* Cross-cultural adaptation and reliability of the pain response to activity and position questionnaire. *Adv Rheumatol Lond Engl* 2019;**59**:53.

17. Takara KS, Alamino Pereira de Viveiro L, Moura PA *et al.* Roland-Morris disability questionnaire is bidimensional and has 16 items when applied to community-dwelling older adults with low back pain. *Disabil Rehabil* 2023;**45**:2526–32.

18. Nagasawa Y, Shibata A, Fukamachi H *et al.* The Psychological Inflexibility in Pain Scale (PIPS): Validity and Reliability of the Japanese Version for Chronic Low Back Pain and Knee Pain. *J Pain Res* 2021;**14**:325–32.

19. Terwee, Prinsen C a. C, Chiarotto A *et al.* COSMIN methodology for evaluating the content validity of patient-reported outcome measures: a Delphi study. *Qual Life Res* 2018;**27**:1159–70.

20. Ustün TB, Chatterji S, Kostanjsek N *et al.* Developing the World Health Organization Disability Assessment Schedule 2.0. *Bull World Health Organ* 2010;**88**:815–23.

21. Symonds, Burton AK, Tillotson KM *et al.* Do attitudes and beliefs influence work loss due to low back trouble. *Occup Med Oxf Engl* 1996;**46**:25–32.

22. Sullivan MJL, Bishop SR, Pivik J. The Pain Catastrophizing Scale: Development and validation. *Psychol Assess* 1995;**7**:524–32.

23. Wicksell RK, Renöfält J, Olsson GL *et al.* Avoidance and cognitive fusion--central components in pain related disability? Development and preliminary validation of the Psychological Inflexibility in Pain Scale (PIPS). *Eur J Pain Lond Engl* 2008;**12**:491–500.

24. Prinsen C a. C, Mokkink LB, Bouter LM *et al.* COSMIN guideline for systematic reviews of patient-reported outcome measures. *Qual Life Res Int J Qual Life Asp Treat Care Rehabil* 2018;**27**:1147–57.

25. Epstein J, Santo RM, Guillemin F. A review of guidelines for cross-cultural adaptation of questionnaires could not bring out a consensus. *J Clin Epidemiol* 2015;**68**:435–41.

26. Mbada CE, Oguntoyinbo OE, Fasuyi FO *et al.* Cross-cultural adaptation and psychometric evaluation of the Yoruba version of Oswestry disability index. *PloS One* 2020;**15**:e0221138.

27. Maki D, Rajab E, Watson PJ *et al.* Cross-cultural translation, adaptation, and psychometric testing of the Roland-Morris disability questionnaire into modern standard Arabic. *Spine* 2014;**39**:E1537-1544.

28. Domazet I, Nemir J, Barl P *et al.* Validation of the Croatian version of the Oswestry Disability Index. *Eur Spine J* 2018;**27**:2814–22.

29. Speksnijder CM, Koppenaal T, Knottnerus JA *et al.* Measurement Properties of the Quebec Back Pain Disability Scale in Patients With Nonspecific Low Back Pain: Systematic Review. *Phys Ther* 2016;**96**:1816–31.

30. Monticone M, Frigau L, Mola F *et al.* The Italian version of the Quebec Back Pain Disability Scale: cross-cultural adaptation, reliability and validity in patients with chronic low back pain. *Eur Spine J* 2020;**29**:530–9.

31. Darnall BD, Sturgeon JA, Cook KF *et al.* Development and Validation of a Daily Pain Catastrophizing Scale. *J Pain* 2017;**18**:1139–49.

32. Chb W, Acc W, Sj M. Meta-analysis of the psychometric properties of the Pain Catastrophizing Scale and associations with participant characteristics. *Pain* 2019;**160**, DOI: 10.1097/j.pain.0000000000001494.

33. Yao M, Zhu S, Tian Z-R *et al.* Cross-cultural adaptation of Roland-Morris Disability Questionnaire needs to assess the measurement properties: a systematic review. *J Clin Epidemiol* 2018;**99**:113–22.

34. Monticone M, Baiardi P, Ferrari S *et al.* Development of the Italian version of the Oswestry Disability Index (ODI-I): A cross-cultural adaptation, reliability, and validity study. *Spine* 2009;**34**:2090–5.

35. Tan B-K, Burnett A, Hallett J *et al.* Back pain beliefs in adolescents and adults in Australasia: A cross-sectional pilot study of selected psychometric properties of paper-based and web-based questionnaires in two diverse countries. *J Back Musculoskelet Rehabil* 2016;**29**:565–74.

36. Maki D, Rajab E, Watson PJ *et al.* Translation, cross-cultural adaptation and psychometric properties of the Back Beliefs Questionnaire in Modern Standard Arabic. *Disabil Rehabil* 2017;**39**:272–80.

37. Amjad F, Mohseni-Bandpei MA, Gilani SA *et al.* Urdu version of Oswestry disability index; a reliability and validity study. *BMC Musculoskelet Disord* 2021;**22**:311.

38. Law KKP, Lee PL, Kwan WW *et al.* Cross-cultural adaptation of Cantonese (Hong Kong) Oswestry Disability Index version 2.1b. *Eur Spine J Off Publ Eur Spine Soc Eur Spinal Deform Soc Eur Sect Cerv Spine Res Soc* 2021;**30**:2670–9.

Supplementary tables

## **Supplementary Table 1. Search Strategy.**

| #1 | reliabilit* OR repeatabilit* OR internal consistenc* OR measurement error* OR test-retest OR inter-rater OR intra-rater OR Reproduciblit* |
| --- | --- |
| #2 | predictive validit* OR structural validit* OR content validit* OR concurrent validit* OR face validit* OR construct validit* OR criterion validit* OR cross-cultural validit* OR concurrent validit* OR criterion validit* OR sensitivity OR specificity |
| #3 | responsiveness OR interpretabilit* |
| #4 | psychometric OR psychometric propert* OR measurement properties OR clinimetric propert* OR COSMIN |
| #5 | #1 OR #2 OR #3 OR #4 |
| #6 | questionnaire* OR scale* OR survey* OR index OR indices OR self-report* OR self-administ* |
| #7 | #5 AND #6 |
| #8 | low back pain OR LBP OR lumbago OR dorsalgia OR sciatic* OR backache OR back-ache OR spinal stenosis OR lumbar stenosis OR disc hernia* OR disc degener* OR spondylol* OR coccydynia OR back injur* |
| #9 | lumbosacral OR lumbo* OR lower back OR low back OR lumbar OR sacrococcyg* OR lumbar vertebra* OR intervertebral disib* |
| #10 | pain OR ache OR agony |
| #11 | #9 AND #10 |
| #12 | #8 OR #11 |
| #13 | older people OR older adult* OR elder* OR older individual* OR frail* OR sarcopeni* OR retired OR elder* OR old age OR centenarian* OR nonagenarian* OR octogenarian* OR septuagenarian* OR aging OR (senior* not ((high school or university or college) adj3 senior*)) OR geronto* OR geriatr* |
| #14 | #12 AND #13 |
| #15 | #7 AND #14 |

## **Supplementary Table 2. Consensus-based Standards for the selection of health Measurement Instruments criteria and rating system for evaluating the content validity of instruments adapted from Mokkink et al. (2018).**

| Name of Instrument | Instrument development study | Content validity study ^1^ | Content validity study ^2^ | Rating of reviewers | Overall rating of an instrument^3^ | Quality of evidence |
| --- | --- | --- | --- | --- | --- | --- |
| Criteria | + / ‐ / ± /? | + / ‐ /± / ? | + / ‐ / ± /? | / ‐ / ± /? | / ‐ / ± | High, moderate, low, very low |
| Relevance  1 Are the included items relevant for the construct of interest?^4^  2 Are the included items relevant for the target population of interest?^4^  3 Are the included items relevant for the context of use of interest?^4^  4 Are the response options appropriate?  5 Is the recall period appropriate? |  |  |  |  |  |  |
| RELEVANCE RATING |  |  |  |  |  |  |
| Comprehensiveness  6 Are all key concepts included? |  |  |  |  |  |  |
| COMPREHENSIVENESS RATING |  |  |  |  |  |  |
| Comprehensibility  7 Are the instrument instructions understood by the population of interest as intended?  8 Are the instrument items and response options understood by the population of interest as intended?  9 Are the instrument items appropriately worded?  10 Do the response options match the question? |  |  |  |  |  |  |
| COMPREHENSIBILITY RATING |  |  |  |  |  |  |
| CONTENT VALIDITY RATING |  |  |  |  |  |  |

Note:^1^ Ratings for the 10 criteria can only be “+ / ‐ /?”. The RELEVANCE, COMPREHENSIVENESS, COMPREHESIBILITY, AND CONTENT VALIDITY ratings can be “+ / ‐ / ± /?”.

^2^ Add more columns if more content validity studies are available.

^3^ If ratings are inconsistent between studies, consider using separate tables for subgroups of studies with consistent results.

^4^ These criteria refer to the construct, population, and context of use of interest in the systematic review.

## **Supplementary Table 3. Criteria for Good Psychometric Properties Adapted from Prinsen et al. (2018).**

| Measurement property | Rating^1^ | Criteria |
| --- | --- | --- |
| Structural validity | + | CTT:  CFA: CFI or TLI or comparable measure >0.95 OR RMSEA  <0.06 OR SRMR <0.082  IRT/Rasch:  No violation of unidimensionality^2^: CFI or TLI or comparable measure >0.95 OR RMSEA <0.06V OR SRMR <0.08^3^  *AND*  No violation of local independence: residual correlations among the items after controlling for the dominant factor < 0.20 OR Q3's < 0.37  *AND*  no violation of monotonicity: adequate looking graphs OR item scalability >0.30  *AND*  adequate model fit:  IRT: χ^2^ >0.01  Rasch: infit and outfit mean squares ≥ 0.5 and ≤ 1.5 OR Z‐ standardized values > ‐2 and |
|  | ? | CTT: Not all information for ‘+’ reported.  IRT/Rasch: Model fit not reported |
|  | – | Criteria for ‘+’ not met |
| Internal consistency | + | At least low evidence^4^ for sufficient structural validity^5^ AND Cronbach's alpha(s) ≥ 0.70 for each unidimensional scale or subscale^6^ |
|  | ? | Criteria for “At least low evidence^4^ for sufficient structural validity^5^” not met |
|  | – | At least low evidence^4^ for sufficient structural validity_5_ AND Cronbach’s alpha(s) < 0.70 for each unidimensional scale or subscale^6^ |
| Reliability | + | ICC or weighted Kappa ≥ 0.70 |
|  | ?  – | ICC or weighted Kappa not reported  ICC or weighted Kappa < 0.70 |
| Measurement error | +  ?  – | SDC or LoA < MIC^5^  MIC not defined  SDC or LoA > MIC^5^ |
| Hypotheses testing for construct validity | + | The result is in accordance with the hypothesis^7^ |
|  | ? | No hypothesis defined (by the review team) |
|  | – | The result is not in accordance with the hypothesis^7^ |
| Cross‐cultural validity\measurement invariance | + | No important differences found between group factors (such as age, gender, language) in multiple group factor analysis OR no important DIF for group factors (McFadden's R2 < 0.02) |
|  | ? | No multiple group factor analysis OR DIF analysis performed |
|  | – | Important differences between group factors OR DIF was found |
| Criterion validity | + | Correlation with gold standard ≥ 0.70 OR AUC ≥ 0.70 |
|  | ? | Not all information for ‘+’ reported |
|  | – | Correlation with gold standard < 0.70 OR AUC < 0.70 |

Note: AUC = area under the curve, CFA = confirmatory factor analysis, CFI = comparative fit index, CTT = classical test theory, DIF = differential item functioning, ICC = intraclass correlation coefficient, IRT = item response theory, LoA = limits of agreement, MIC = minimal important change, RMSEA: Root Mean Square Error of Approximation, SEM = Standard Error of Measurement, SDC = smallest detectable change, SRMR: Standardized Root Mean Residuals, TLI = Tucker‐Lewis index.

^1^ “+” = sufficient, “–” = insufficient, “?” = indeterminate.

^2^ Unidimensionality refers to a factor analysis per subscale, while structural validity refers to a factor analysis of a (multidimensional) patient‐reported outcome measure.

^3^ To rate the quality of the summary score, the factor structures should be equal across studies.

^4^ As defined by grading the evidence according to the GRADE approach.

^5^ This evidence may come from different studies.

^6^ The criteria ‘Cronbach alpha < 0.95’ was deleted, as this is relevant in the development phase of a PROM and not when evaluating an existing PROM.

^7^ The results of all studies should be taken together, and it should then be decided if 75% of the results are in accordance with the hypotheses

## **Supplementary Table 4. Study results and ratings of values on psychometric properties (according to GRADE approach).**

| **Psychometric Property** | **Instrument** | **Reference** | **Risk of Bias** | **Sample Size** | **Results (rating)** | **Quality of Evidence (reasons)** |
| --- | --- | --- | --- | --- | --- | --- |
| **Structural validity** | ODI | Jenk et al., 2022 | Inadequate | 193 | RMSEA=0.068; SRMR=0.0588 (+) | **Very low** (downgrading by three levels of risk of bias; only one study of inadequate quality) |
|  | QBPDS | Jenk et al., 2022 | Inadequate | 193 | CFI=0.88; TLI=0.87; RMSEA=0.18; SRMR=0.09 (-) | **Very low** (downgrading by three levels of risk of bias; only one study of inadequate quality) |
|  | RMDQ | Jenk et al., 2022 | Inadequate | 193 | RMSEA=0.057; SRMR=0.121 (+) | **Low** (downgrading by two levels of risk of bias; two studies of inadequate quality) |
|  |  | Takara et al., 2023 | Inadequate | 528 | RMSEA=0.037 (+) |  |
|  | RMDQ subgroup analysis (acute and subacute NSLBP) | Jenk et al., 2022 | Inadequate | 193 | RMSEA=0.057; SRMR=0.121 (+) | **Very low** (downgrading by three levels of risk of bias; only one study of inadequate quality) |
|  | PIPS | Nagasawa et al., 2021 | Adequate | 94 | CFI=0.97; RMSEA=0.06 (+) | **Low** (downgrading by one level of risk of bias, one level of imprecision and one level of indirectness; only one study of adequate quality, small sample (n=94), sample with CNSLBP and knee pain) |
| **Hypothesis testing for construct validity** | ODI | Hicks et al., 2009 | Doubtful | 107 | Evaluating the relationship between ODI and QBPDS in terms of their pain severity and physical function: *P* < 0.001 (+) | **Moderate** (downgrading by one level of risk of bias; two studies of very good quality and doubtful quality, respectively) |
|  |  | Jenk et al., 2022 | Very good | 193 | ODI met at least 75% of the hypotheses of construct validity (+) |  |
|  | ODI subgroup analysis (acute and subacute NSLBP) | Jenk et al., 2022 | Very good | 193 | ODI met at least 75% of the hypotheses of construct validity (+) | **High** (no concern) |
|  | QBPDS | Hicks et al., 2009 | Doubtful | 107 | Evaluating the relationship between ODI and QBPDS in terms of their pain severity and physical function: *P* < 0.001 (+) | **Moderate** (downgrading by one level of risk of bias; two studies of very good quality and doubtful quality, respectively) |
|  |  | Jenk et al., 2022 | Very good | 193 | QBPDS met at least 75%  of the hypotheses of construct validity (+) |  |
|  | QBPDS  subgroup analysis (acute and subacute NSLBP) | Jenk et al., 2022 | Very good | 193 | QBPDS met at least 75%  of the hypotheses of construct validity (+) | **High** (no concern) |
|  | RMDQ | Jenk et al., 2022 | Very good | 193 | RMDQ met at least 75% of the hypotheses of construct validity (+) | **High** (no concern) |
|  | BBQ | Tingulstad et al., 2019 | Doubtful | 116 | The correlation analysis confirmed 75% of the predefined hypotheses, indicating a good construct validity  with FABQ-PA: the correlation coefficient value = -0.57, with PCS: the correlation coefficient value = -0.45, with RMDQ: the correlation coefficient value = -0.45, with NRS: the correlation coefficient value = -0.14 (+) | **Low** (downgrading by two levels of risk of bias; only one study of doubtful quality) |
| **Internal consistency** | ODI | Bayar et al., 2003 | Doubtful | 29 | Cronbach’s Alpha = 0.722 (baseline)/0.717 (test) (+) | **High** (no concern) |
|  |  | Jenk et al., 2022 | Very good | 193 | Cronbach’s Alpha = 0.86 (+) |  |
|  | ODI subgroup analysis (acute and subacute NSLBP) | Jenk et al., 2022 | Very good | 193 | Cronbach’s Alpha = 0.86 (+) | **High** (no concern) |
|  | QBPDS | Jenk et al., 2022 | Very good | 193 | Cronbach’s Alpha = 0.94 (+) | **High** (no concern) |
|  | RMDQ | Bayar et al., 2003 | Doubtful | 29 | Cronbach’s Alpha = 0.86 (+) | **High** (no concern) |
|  |  | Jenk et al., 2022 | Very good | 193 | Cronbach’s Alpha = 0.89 (+) |  |
|  | RMDQ subgroup analysis (acute and subacute NSLBP) | Jenk et al., 2022 | Very good | 193 | Cronbach’s Alpha = 0.89 (+) | **High** (no concern) |
|  | WHODAS 2.0 | Agnieszka et al., 2020 | Doubtful | 65 | Cronbach’s Alpha = 0.92 (+) | **Moderate** (downgrading by one level of imprecision; small sample (n=65)) |
|  | BBQ | Teixeira et al., 2020 | Inadequate | 26 | Cronbach’s Alpha = 0.70 (+) | **Low** (downgrading by two levels of risk of bias; one study of doubtful quality) |
|  |  | Tingulstad et al., 2019 | Doubtful | 116 | Cronbach’s Alpha = 0.82 (+) |  |
|  | CAS-D-65+ | Quint et al., 2011 | Doubtful | 68 | Cronbach’s Alpha = 0.87-0.92 (+) | **Moderate** (downgrading by one level of imprecision; small sample (n=68)) |
|  | PIPS | Nagasawa et al., 2021 | Doubtful | 94 | Cronbach’s Alpha = 0.85 (+) | **Very low** (downgrading by two levels of risk of bias, one level of imprecision and indirectness, respectively; one study of doubtful quality, small sample (n=94); sample with CNSLBP and knee pain) |
|  | FRI | Bayar et al., 2004 | Inadequate | 76 | Cronbach’s Alpha=0.921 (test)/ 0.901 (retest) (+) | **Moderate**(downgrading by one level of imprecision; small sample (n=76)) |
| **Reliability** | ODI | Bayar et al., 2003 | Doubtful | 29 | Test-retest Reliability: ICC=0.93 (+) | **Moderate** (downgrading by one level of risk of bias; two studies of doubtful quality) |
|  |  | Hicks et al., 2009 | Doubtful | 107 | Test-retest Reliability: ICC = 0.92 (+) |  |
|  |  | Jenk et al., 2022 | Adequate | 214 | Test-retest Reliability: ICC= 0.89 (+) |  |
|  | ODI subgroup analysis (acute and subacute NSLBP) | Jenk et al., 2022 | Adequate | 214 | Test-retest Reliability: ICC= 0.89 (+) | **Moderate** (downgrading by one level of risk of bias; one study of adequate quality) |
|  | QBPDS | Hicks et al., 2009 | Doubtful | 107 | Test-retest Reliability: ICC= 0.92 (+) | **Moderate** (downgrading by one level of risk of bias; only one study of adequate quality) |
|  |  | Jenk et al., 2022 | Adequate | 214 | Test-retest Reliability: ICC= 0.84 (+) |  |
|  | QBPDS subgroup analysis (acute and subacute NSLBP) | Jenk et al., 2022 | Adequate | 214 | Test-retest Reliability: ICC= 0.84 (+) | **Moderate** (downgrading by one level of risk of bias; only one study of adequate quality) |
|  | RMDQ | Jenk et al., 2022 | Adequate | 214 | Test-retest Reliability: ICC= 0.85 (+) | **Moderate** (downgrading by one level of risk of bias; only one study of adequate quality) |
|  | WHODAS 2.0 | Agnieszka et al., 2020 | Doubtful | 65 | Test-retest Reliability: ICC= 0.928 (+) | **Very low** (downgrading by two levels of risk of bias and one level of imprecision; only one study of doubtful quality, small sample (n=65)) |
|  | BBQ | Teixeira et al., 2020 | Inadequate | 26 | Test-retest Reliability: ICC = 0.74 (+) | **Moderate** (downgrading by one level of risk of bias; only one study of adequate quality) |
|  |  | Tingulstad et al., 2019 | Adequate | 116 | Test-retest Reliability: ICC = 0.71 (+) |  |
|  | CAS-D-65+ | Quint et al., 2011 | Inadequate | 68 | Test-retest Reliability: ICC: 0.67 to 0.70 (-) | **Very low**(downgrading by three levels of risk of bias and one level of imprecision; only one study of inadequate quality, small sample (n=68)) |
|  | PCS | Lopes et al., 2015 | Doubtful | 131 | Intra-rater Reliability: Kappa = 0.80±0.01, ICC = 0.88 (+) | **Low** (downgrading by two levels of risk of bias; only one study of doubtful quality) |
|  | FRI | Bayar et al., 2004 | Doubtful | 76 | Test-retest Reliability: ICC= 0.913 (+) | **Very low** (downgrading by two levels of risk of bias and one level of imprecision; only one study of doubtful quality, small sample (n=76)) |
|  | PRAP | de Carvalho et al., 2019 | Inadequate | 36 | Intra-rater Reliability: Kappa = 0.50 to 1.00 (-) | **Very low** (downgrading by three levels of risk of bias and two levels of imprecision; one study of inadequate quality, small sample (n=36)) |
| **Measurement error** | ODI | Jenk et al., 2022 | Adequate | 214 | SDC = 19.11 ＞ MIC = 10 (-) | **Moderate** (downgrading by one level of risk of bias; only one study of adequate quality) |
|  | QBPDS | Jenk et al., 2022 | Adequate | 214 | SDC = 23.58 ＞ MIC = 20 (-) | **Moderate** (downgrading by one level of risk of bias; only one study of adequate quality) |
|  | RMDQ | Jenk et al., 2022 | Adequate | 214 | SDC = 6.87 ＞ MIC = 5 (-) | **Moderate** (downgrading by one level of risk of bias; only one study of adequate quality) |
| **Criterion validity** | ODI | Bayar et al., 2003 | Doubtful | 29 | Correlation with VAS = 0.53; Correlation with RMDQ = 0.66 (-) | **Very low** (downgrading by two levels of risk of bias and two levels of imprecision; only one study of doubtful quality, small sample (n=29)) |
|  | RMDQ | Bayar et al., 2003 | Doubtful | 29 | Correlation with VAS = 0.46 (-) | **Very low** (downgrading by two levels of risk of bias and two levels of imprecision; only one study of doubtful quality, small sample (n=29)) |
|  | PIPS | Nagasawa et al., 2021 | Doubtful | 94 | Correlation with acceptance and Action Questionnaire-II = 0.58; Correlation with Cognitive Fusion Questionnaire = 0.45 (-) | **Very low** (downgrading by two levels of risk of bias and one level of imprecision; only one study of doubtful quality, small sample (n=94)) |
|  | FRI | Bayar et al., 2004 | Adequate | 76 | Correlation with NRS = 0.701 (+) | **Low** (downgrading by one level of risk of bias and one level of imprecision; only one study of adequate quality, small sample (n=76)) |
| **Responsiveness** | ODI | Jenk et al., 2022 | Very good | 214 | AUC=0.72 (+) | **High** (no concern) |
|  | QBPDS | Jenk et al., 2022 | Very good | 214 | AUC=0.75 (+) | **High** (no concern) |
|  | RMDQ | Jenk et al., 2022 | Very good | 214 | AUC=0.75 (+) | **High** (no concern) |

Note: AUC = The area under the curve; BBQ = Back Beliefs Questionnaire; CAS-D-65+ = Catastrophizing Avoidance Scale D-65+; CFA = confirmatory factor analysis; CFI = comparative fit index; CI = confidence interval; CNSLBP = chronic non-specific low back pain; EFA = exploratory factor analysis; FRI = Functional Rating Index; GFI = goodness of fit index; ICC = Intraclass Correlation Coefficient; KMO = Kaiser–Meyer–Olkin; NNFI = non-normed fit index; NRS = Numeric Rating Scale; NSLBP = non-specific low back pain; ODI = Oswestry Disability Index; PCS = Pain Catastrophizing Scale; PIPS = Psychological Inflexibility in Pain Scale; PRAP = Pain Response to Activity and Positioning questionnaire; QBPDS = Quebec Back Pain Disability Scale; r = Pearson product moment correlation coefficients; RMDQ = Roland-Morris Disability Questionnaire; RMSEA = root-mean-square error of approximation; SDC = smallest detectable change; SRMR = standardized root-mean-square residual; TLI = Tucker-Lewis index; VAS = visual analogue scale; WHODAS 2.0 = The 36-Item World Health Organization Disability Assessment Schedule 2.0.

“+” = Sufficient rating; “?” = Indeterminate rating; “-” = Insufficient rating; “±” = Inconsistent rating; High = High level of confidence in overall ratings; Moderate = Moderate level of confidence in overall ratings; Low = Low level of confidence in overall ratings; Very Low = Very low level of confidence in overall ratings.

## **Supplementary Table 5. Details of Consensus-based Standards for the selection of health Measurement Instruments Risk of Bias.**

| Study | Structural validity | Hypotheses testing for construct validity | Internal consistency | Reliability | Measurement error | Criterion validity | Responsiveness |
| --- | --- | --- | --- | --- | --- | --- | --- |
| (Agnieszka et al., 2020) |  |  | 1(doubtful), 2(adequate), 3(doubtful), 4(very good), 5(very good),6(-)  Final: doubtful | 1(adequate),2(adequate),3(adequate),4(very good),5(adequate), 6(doubtful), 7(adequate), 8(doubtful),9(adequate),10(doubtful),11(very good),12(very good), 13(very good),14(adequate)  Final: doubtful |  |  | 10d: 1(very good), 2(adequate), 3(very good)  Final: adequate |
| (Bayar et al., 2003) |  |  | 1(doubtful), 2(inadequate), 3(doubtful), 4(doubtful), 5(doubtful),6(-)  Final: doubtful | 1(adequate),2(adequate), 3(inadequate),4(very good), 5(adequate),6(very good), 7(adequate),8(very good), 9(adequate),10(very good), 11(adequate),12(very good), 13(doubtful),14(adequate)  Final: doubtful |  | 1(adequate), 2(adequate), 3(doubtful)  Final: doubtful |  |
| (Bayar et al., 2004) |  | 1(very good), 2(very good), 3(very good), 4(doubtful), 5(adequate), 6(adequate), 7(doubtful)  Final: doubtful | 1(doubtful), 2(doubtful), 3(doubtful), 4(very good), 5(inadequate), 6(inadequate)  Final: inadequate | 1(adequate),2(doubtful),3(doubtful),4(very good),5(adequate), 6(doubtful),7(doubtful),8(doubtful),9(doubtful),10(doubtful),11(adequate),12(inadequate),13(inadequate),14(adequate)  Final: doubtful |  | 1(adequate), 2(adequate), 3(adequate)  Final: adequate |  |
| (Carvalho et al., 2019) |  |  |  | 1(adequate),2(doubtful),3(doubtful),4(inadequate),5(doubtful),6(doubtful),7(doubtful),8(doubtful),9(doubtful), 10(doubtful),11(adequate),12(inadequate),13(doubtful),14(adequate)  Final: inadequate |  |  |  |
| (Lopes et al., 2015) |  | 1(very good), 2(very good), 3(very good), 4(very good), 5(very good), 6(adequate), 7(doubtful)  Final: doubtful |  | 1(adequate),2(doubtful),3(Very good),4(very good),5(doubtful), 6(doubtful),7(doubtful),8(doubtful),9(doubtful),10(doubtful),11(adequate),12(very good),13(very good), 14(adequate)  Final: doubtful |  |  |  |
| (Nagasawa et al., 2021) | 1(very good),2(adequate),3(adequate),4(very good)  Final: adequate | 1(very good),2(very good),3(very good),4(very good),5(adequate),6(adequate),7(doubtful)  Final: doubtful | 1(doubtful),2(very good),3(doubtful),4(doubtful),5(doubtful),6(-)  Final: doubtful | 1(adequate),2(adequate),3(Very good),4(very good),5(adequate),6(very good),7(adequate),8(very good),9(adequate),10(very good),11(doubtful),12(very good),13(doubtful),14(adequate)  Final: doubtful |  | 1(adequate), 2(adequate), 3(doubtful)  Final: doubtful |  |
| (Quint S et al., 2011) |  |  | 1(doubtful),2(adequate),3(doubtful),4(doubtful),5(doubtful),6(-)  Final: doubtful | 1(adequate),2(doubtful),3(adequate),4(inadequate),5(doubtful),6doubtful),7(adequate),8(doubtful),9(adequate),10(doubtful),11(doubtful),12(inadequate),13(inadequate),14(adequate)  Final: inadequate |  |  |  |
| (Teixeira et al., 2020) |  |  | 1(doubtful),2(inadequate),3(doubtful),4(very good),5(inadequate),6(inadequate)  Final: inadequate | 1(adequate),2(doubtful),3(inadequate),4(inadequate),5(doubtful),6(doubtful),7(adequate),8(doubtful),9(adequate),10(doubtful),11(adequate),12(inadequate),13(doubtful),14(adequate)  Final: inadequate |  |  |  |
| (Tingulstad et al., 2019) |  | 1(very good),2(very good),3(very good),4(very good),5(adequate),6(adequate),7(doubtful)  Final: doubtful | 1(doubtful),2(very good),3(doubtful),4(very good),5(very good),6(-)  Final: doubtful | 1(adequate),2(adequate),3(very good),4(very good),5(adequate),6(very good),7(adequate),8(very good),9(adequate),10(very good),11(adequate),12(very good),13(very good),14(adequate)  Final: adequate |  |  |  |

Note: NA = Not assessed.

## **Supplementary Table 6. Overall quality of content validity and quality of evidence per instrument.**

| Instrument | Overall quality of content validity^a^ | Quality of evidence^b^ |
| --- | --- | --- |
| ODI | + | Low |
| QBPDS | + | Low |
| RMDQ | + | Low |
| WHODAS 2.0 | - | Low |
| BBQ | ± | Low |
| CAS-D-65+ | ± | Low |
| PCS | ± | Very low |
| PIPS | ± | Low |
| FRI | + | Low |
| PRAP | + | Low |

Note: ^a^ The quality of content validity (relevance, comprehensiveness, and comprehensibility) per study and content of instrument was rated using the criteria for good content validity (Terwee et al., 2018); “+” = sufficient rating; “-” = insufficient rating; “?” = indeterminate rating; “±” = inconsistent rating

^b^ The quality of evidence for content validity was rated using the modified GRADE approach (Terwee et al., 2018); high = high-level of confidence; moderate = moderate-level of confidence; low = low-level of confidence; very low = very low-level of confidence;

Abbreviations: BBQ = Back Beliefs Questionnaire; CAS-D-65+= Catastrophizing Avoidance Scale D-65+; FRI = Functional Rating Index; ODI = Oswestry Disability Index; PCS = Pain Catastrophizing Scale; PIPS = Psychological Inflexibility in Pain Scale; PRAP = Pain Response to Activity and Positioning questionnaire; QBPDS = Quebec Back Pain Disability Scale; RMDQ = Roland-Morris Disability Questionnaire; WHODAS 2.0 = The 36-Item World Health Organization Disability Assessment Schedule 2.0.

## **Supplementary Fig.1. A PRISMA flow diagram of the study selection process.**


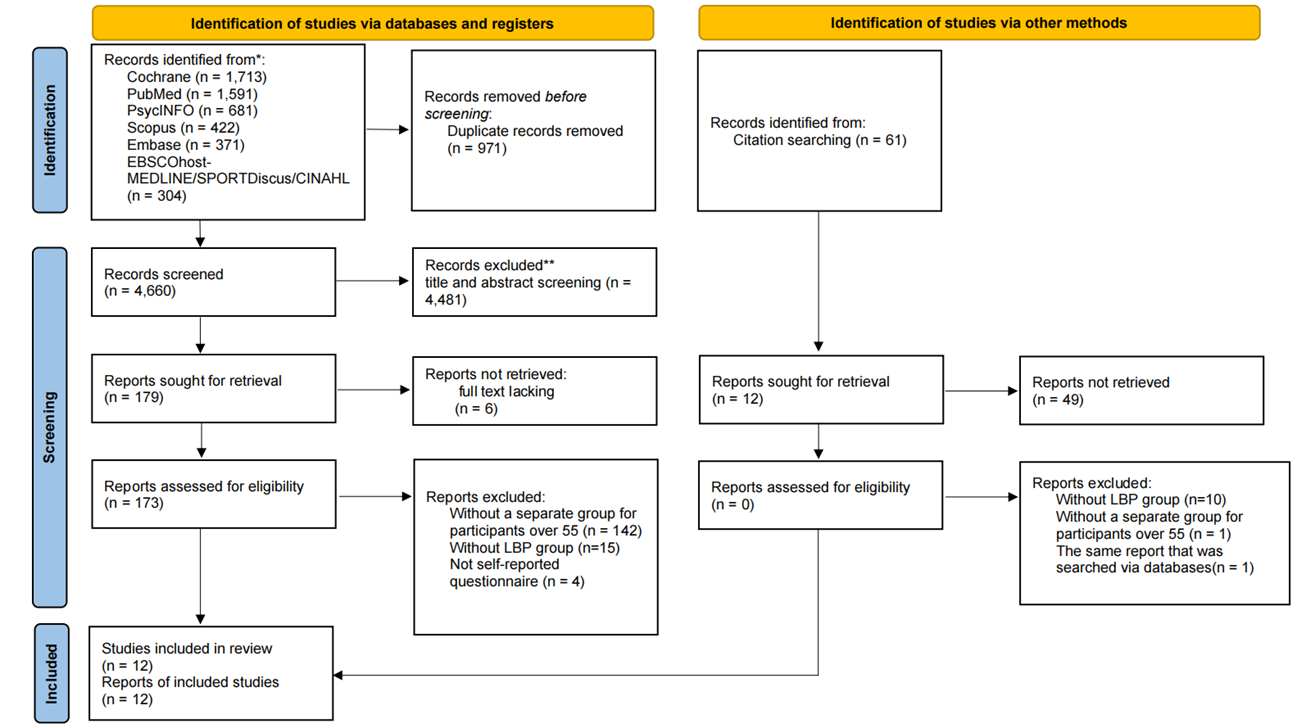

Supplement: aa-24-1148-File003_afaf045 [file aa-24-1148-file003_afaf045.docx]
